# Video Activity Recognition: State-of-the-Art

**DOI:** 10.3390/s19143160

**Published:** 2019-07-18

**Authors:** Itsaso Rodríguez-Moreno, José María Martínez-Otzeta, Basilio Sierra, Igor Rodriguez, Ekaitz Jauregi

**Affiliations:** 1 Department of Computer Science and Artificial Intelligence, University of the Basque Country, Manuel Lardizabal 1, 20018 Donostia-San Sebastián, Spain; 2 Department of Computer Languages and Systems, University of the Basque Country, Manuel Lardizabal 1, 20018 Donostia-San Sebastián, Spain

**Keywords:** activity recognition, computer vision, optical flow, deep learning

## Abstract

Video activity recognition, although being an emerging task, has been the subject of important research efforts due to the importance of its everyday applications. Surveillance by video cameras could benefit greatly by advances in this field. In the area of robotics, the tasks of autonomous navigation or social interaction could also take advantage of the knowledge extracted from live video recording. The aim of this paper is to survey the state-of-the-art techniques for video activity recognition while at the same time mentioning other techniques used for the same task that the research community has known for several years. For each of the analyzed methods, its contribution over previous works and the proposed approach performance are discussed.

## 1. Introduction

Activity recognition consists of identifying some actions from a series of observations. This field has caught the interest of many researchers since the 1980s due to the number of applications for which it is useful, such as medicine [1,2], human–computer interaction [3,4], surveillance [5,6] or sociology [7,8]. For instance, in surveillance [9,10], the automatic detection of suspicious actions would allow for launching a warning and taking measures against any danger. Another example is the use of activity recognition for rehabilitation [11], recognizing the action the patients are performing and having the ability to determine if it is right or not. One of the main techniques used for activity recognition is computer vision, namely video-based activity recognition. Visual video features provide basic information for video events or actions.

The task of tracking and understanding what is happening in a video can be very challenging. Many attempts have been made lately using different techniques [12,13,14] such as optical flow [15,16], Hidden Markov Models (HMM) [17,18,19] or, more recently, deep learning [20,21]. Furthermore, apart from using multiple techniques, many different scenarios are being considered, single action recognition [22,23], group tracking [24,25], etc.

However, despite remarkable progress, the advances achieved so far do not meet high accuracy standards and the correct realization of this task in some areas, such as video surveillance, is still an open research issue.

In the analysis of a video content, many different functionalities can be implemented. One of the simplest ways to detect motion regarding a fixed background is Video Motion Detection [26,27,28]. Video tracking [29,30] is more challenging than the previous approach and can be very time consuming, due to the amount of data that a video contains. The aim of video tracking is to associate target objects in consecutive video frames, which can be especially difficult if the objects are moving fast in relation to the frame rate. If object recognition techniques are needed (a challenging problem in its own), further complexity is added. On the contrary, the human brain seems to have the ability to recognize human actions perfectly. This aptitude is not just related to acquired knowledge, but also to logical reasoning and the capability of extracting relevant information from context. Based on this, the integration of commonsense reasoning [31,32] and contextual knowledge [33] has been proposed.

Hence, action recognition involves the classification of different actions from videos, a sequence of frames, taking into account as well the fact that the action could not be performed during the entire video. Although it seems an extension of image classification tasks, as it has been mentioned before, the progress for video classification has been slower due to various reasons:

- Apart from spatial information, temporal context across frames is also required.
- Huge computational cost.
- Datasets are more limited, due to the difficulty to collect, annotate and store videos.

**Figure 1:**
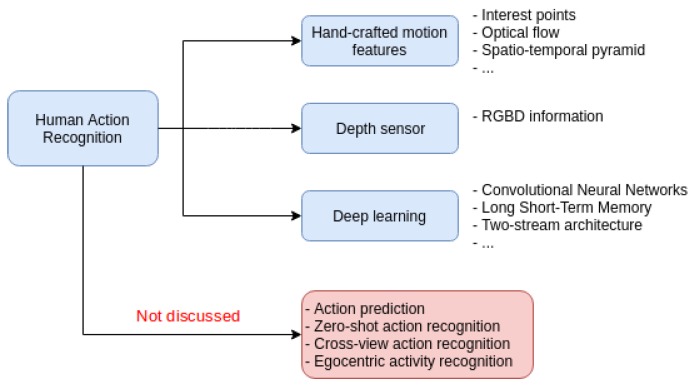
Summary diagram.

Throughout this paper, several techniques applied for video activity recognition are mentioned, as well as the latest contributions made in the field. In addition, as a final note, some of the databases used for this topic are presented along with the results of the latest contributions using them. In Figure 1, a diagram showing the techniques explained and other tasks related to this subject but which are not discussed in this review are indicated.

This review focuses on a specific area of Human Action Recognition, to keep the discussion simple. Only action recognition from a whole video recorded from a fixed position is considered in this paper, as we think this problem setup is the entrance gate to the analysis of other more complex situations, as those presented in the bottom part of Figure 1. At the same time, the complexity level of the problem considered in this review is high enough to deserve a dedicated survey. For the sake of completeness, we will briefly review the main characteristics of the situations shown in Figure 1 but not covered here. In action prediction, instead of recognizing the action that is happening in the video, the objective is to guess the action that will occur in an incomplete video. The zero-shot action recognition problem consists of training a model to classify videos of categories that have no instances in the training set, which means that there are no instances of certain classes that are going to appear in the test set. To address this issue, complementary information of invisible classes is assumed in the form of attribute vectors that describe each class. In the cross-view action recognition, there are different points of view in the scene when the action is occurring. There are other variations such as egocentric activity recognition that consists of recognizing actions from egocentric videos [34].

The survey is centered in action recognition methods for videos that are recorded in third person and the whole action occurs inside the video. Although different information can be extracted from the videos and there are articles mentioned that also use extra information such as depth sensors’ information, all the presented methods have these two characteristics in common. The methods that are explained use databases with the characteristics of the ones presented in Section 3. Although there are previous reviews on video action recognition [12,35,36], as it is a subject that is continuously progressing, it is always necessary to have a survey that collects the latest contributions. Our review, apart from mentioning articles that others have not been able to collect since they have been published later, also deals with older articles that have served as reference for later methods.

## 2. Used Techniques

As activity recognition has been an active research area lately, there have been many different approaches to deal with this problem. Throughout the survey, some of these are introduced, starting with simpler approaches and finishing with the newest contributions to the field. The proposed methods that try to solve this problem that are referred to in this paper could be separated into three main groups: methods using hand-crafted motion features, depth information based methods and deep learning based methods. Strictly speaking, these three areas are somehow interrelated and depth sensors features could lie under the hand-crafted or deep learning categorization. For a long time, computer vision has focused on data recorded from RGB (visible light) cameras, especially in the case of videos. Depth sensors have started to be used in the field of video analysis in more recent times and this is the reason why we feel it deserves a separate section.

First, hand-crafted motion features methods are explained. In these methods, some interesting features are obtained from the raw pixels of the video frames and then these features are used to perform the recognition. Second, depth information based methods are analyzed, which use depth maps as extra information. Third, deep learning methods are presented, which, unlike hand-crafted methods, achieve the features for the recognition automatically. Throughout the document, several methods that combine some of these three modalities are also presented.

### 2.1. Methods Using Hand-Crafted Motion Features

This document focuses on video-based activity recognition, in which the representation of visual and temporal information becomes important. There are several ways to extract visual features, both static image features and temporal visual features, and then use them to perform the recognition. Temporal visual features are a combination of static image features and time information, so, through these features, temporal video information is achieved. Key-frame [37,38], bag-of-words (BoW) [39,40], interest points [41,42] and motion based approaches [43,44,45,46] are types of representations that can be obtained from a video. *Key-frame* based approaches, as the name indicates, consist of detecting the key-frames of the video which would be used for classification; *BoW* based approaches represent the frames of the video segments over a vocabulary of visual features; *interest points* based approaches focus on simply selecting a specific set of points or pixels for the classification and, to finish, *motion* based approaches focus on the movement along the video. Throughout this section, only motion based approaches are analyzed.

In [47], the authors use a temporal template as the basis of their representation, continuing with their approach presented in [48]. This temporal template consists of a static vector-image where the value of the vector at each point represents a function of the motion properties at the corresponding spatial location in an image sequence. They explore their representation with a simple two component version of the template:

- The first value indicates the presence of motion and where it occurs by a binary motion-energy image (MEI). Being D(x,y,t) a binary image sequence and *r* the value that defines the temporal extent of a movement, the binary image is defined this way:
  (1) $$E_{r}\left(x,y,t\right)=\overset{r-1}{\underset{i=0}{⋃}}D\left(x,y,t-i\right).$$
- The second value is a scalar-valued image where intensity is a function of recency of motion of the sequence, represented by a motion-history image (MHI) which indicates how the image is moving. $H_{r}$ represents the temporal history of motion at each point, where recently moved pixels are brighter:
  (2) $$H_{r}\left(x,y,t\right)=\left\{\begin{array}{cc} r, & \mathrm{if}D(x,y,t)=1,\\ max(0,H_{r}\left(x,y,t-1\right)-1), & \mathrm{otherwise}. \end{array}\right.$$

Then, a recognition method is developed, which matches these temporal templates against stored instances of known actions. They also present a recognition method to automatically perform temporal segmentation being invariant to linear changes in speed.

The authors of [49] demonstrate that local measurements in terms of spatio-temporal interest points (local features) can be used to recognize complex motion patterns. As these features, which capture local motion events in videos, can be adapted to size, frequency and velocity of moving patterns, the resulting video representations are stable with respect to the corresponding transformations. To represent motion patterns, they use local space-time features [50] and to detect local features they construct, using Gaussian convolution, its scale-space representation. Then, they explore the integration of local space-time features with Support Vector Machines (SVM) classifier [51,52], used in many visual pattern recognition methods [53,54], and apply the resulting approach to the recognition of human actions. In addition, for the purpose of evaluation, the authors introduce a new video database containing 2391 clips of six human-actions performed by 25 people in four scenarios.

In [55], the authors present a hybrid hierarchical model, inspired by [56], where video sequences are represented as collections of spatial and spatio-temporal features. These features are achieved by extracting both static and dynamic interest points and the model is able to combine static and motion image features, as well as performing categorization of human actions in a frame-by-frame basis. Motion features are extracted as in [40]. They show that using static and dynamic features together is better than using just a single feature type.

Laptev et al. [42] contribute to the recognition of realistic videos and use movie scripts for automatic annotation of human actions in videos. Due to the achievements in image classification [57,58,59,60], they employ spatio-temporal features and spatio-temporal pyramids, extending spatial pyramids of [58]. Interests points are detected as in [50] using a space-time extension of the Harris operator [61]. Then, a multi-scale approach is used and features at multiple levels are extracted. For classification, they use a nonlinear SVM with a multi-channel Gaussian kernel [60]. Apart from the action recognition task, their main contribution consists of automatically annotating human actions with the use of movie scripts and getting videos with more realistic characteristics.

Visual features such as edges, corners, interest points, etc. can be used to form a more complicated feature called optical flow. The optical flow methods try to calculate the motion between two image frames which are taken at times *t* and t+Δt at every position, assuming that the intensity of objects does not change during the movement I(x,y,t)=I(x+Δx,y+Δy,t+Δt). Expanding that equation using the Taylor Series Expansion [62] and further calculations, this equation is obtained:

(3) $$I_{x}V_{x}+I_{y}V_{y}=-I_{t}$$

or

(4) $$\nabla I^{T}\cdot \vec{V}=-I_{t}.$$

The solution, the optical flow, is the value of $\vec{V}$. Some approaches are given in the calculation of optical flow due to the fact that there are two unknowns in the equation. In this part, several methods that have made use of this feature and its variations are presented.

The authors of [63] present a method to recognize human actions observing them from a far field of view, but they also test their model with normal resolution datasets, such as Weizmann [64]. They use Histograms of Oriented Gradients (HOG) for human pose representations, first introduced in [65] and successfully applied in multiple action recognition methods [66,67,68,69]. They also use a time series of Histogram of Oriented Optical Flow (HOOF) to characterize human motion. To get a subset of discriminantly informative principal components (PCs), an extension of Supervised Principal Component Analysis (SPCA) [70] technique is used, which tries to select a subset of PCs in order to best separate samples projected from different classes. This step significantly speeds up the run-time of recognition without sacrificing accuracy. A multi-class Support Vector Machine (SVM) classifier is trained for action classification. The classifier prediction is made by a collection of one-against-one SVM classifiers, as in the implementation of [71].

In [46], inspired by the success of histograms of features in object recognition, the authors propose the representation of each frame with the use of HOOF features, which are independent from the scale of the moving person and to the direction of motion. These histograms are created by computing optical flow at every frame and binning the vectors according to each primary angle. To classify HOOF time-series, they posit a generalization of the Binet–Cauchy kernels [72] to nonlinear dynamical systems (NLDS), as the data that represents, for instance, that the histogram time series is non-Euclidean and needs to be modeled with nonlinear dynamical systems. The generalization is done by using a Mercer kernel [73] on the output space. The Binet–Cauchy kernels are used for NDLS to perform the activity recognition and proposed HOOF features as outputs of NLDS.

The authors of [45] introduce a motion descriptor based on direction of optical flow. In their method, interest silhouettes are subtracted from the background (used dataset provides foreground masks [64]) and optical flow is computed using the Lucas–Kanade algorithm [74]. Then, before computing a direction histogram, the window is divided into eight regions. To represent the distribution of optical flow direction, they use a histogram, segmenting the direction of optical flow into eight bins. To create the motion vector, they concatenate a direction histogram of optical flow in every region. They also smooth the motion vectors to reduce motion variation and noise, and then these vectors are used for classification. K-means clustering [75] is first used to group similar postures and then the classification is done by a K-NN classifier. Niebles et al. [55] also used clustering but with a bag-of-words model instead of motion.

Due to the demonstration of dense trajectories being efficient video representations, in [76], their performance is improved by using camera motion to correct them. The estimation of camera motion is done by matching feature points between frames using SURF (speeded up robust features) descriptors [77] and dense optical flow [78]. A human detector [79,80] is used to remove inconsistent matches generated because of the differences of human and camera motions and, in addition, background trajectories are also removed. Motion-based descriptors, such as HOF (histogram optical flow), are significantly improved by this.

In [81], the authors propose a generic temporal video segment representation method for action recognition based on optical flow concept [62], with the idea that, to deal with a video-based action recognition problem, temporally represented video information is needed. In their approach, for feature detection, the Shi–Tomasi algorithm is used [82], which is based on Harris corner detector [61], and, to estimate optical flow, the Lucas–Kanade algorithm [74] is computed. For each selected frame of the video, optical flow vectors are grouped according to their angular features. Being an optical flow histogram the most common method of optical flow based video representation, they enrich these approaches by a novel velocity concept, Weighted Frame Velocity. This concept refers to the velocity of cumulative angular grouping of a temporal video segment, which represents the motion of the frames more descriptively. Similarities in the histogram do not always mean that there are similarities in the motion, so, instead of using a histogram based approach as in [46,83,84,85], vectors are grouped with respect to their angular characteristics and then summed and integrated with the new velocity concept.

The authors of [15] propose a local descriptor built by optical flow vectors along the edges of the action performers. First, a foreground extraction is done by a Gaussian Mixture Model (GMM) based method [86] and optical flow based technique [62] in order to segment the region of interest. To represent the segmented objects, optical flow based feature vectors are computed along the boundary using Horn and Schunck algorithm [62] based optical flow extraction technique. This way, shape and instantaneous velocity information extracted from the boundaries of the action performers are incorporated in the feature set. These features are then used to feed a multi-class SVM classifier.

In [87], human activities are recognized using background subtraction, HOG features and Back-Propagation Neural Network (BPNN) classifier. In this approach, background estimation is performed at first, using mean filter to obtain the background and areas of the image containing important information. Afterwards, in order to extract features to describe human motion, a histogram of oriented gradients (HOG) [65] descriptor is used, with the idea that local shape information can be completely described by intensity gradients or edge directions. Finally, a BPNN is used to perform the final classification.

In Table 1, a summary of the explained methods using hand-crafted motion features is presented.

### 2.2. Depth Information Based Methods

The interest of applying depth data captured from depth cameras for the action recognition problem has grown due to the advances of imaging technology in capturing depth information in real time, such as Microsoft Kinect [92] and Intel Realsense [93]. In the past few decades, research of human action recognition has mainly concentrated on video sequences captured by traditional RGB cameras, but, thanks to the advances in imaging techniques, RGBD sensors are able to capture color image sequences together with depth maps in real time. Depth images are insensitive to changes in lighting conditions and provide additional body shape and motion information that can help with distinguishing actions that generate similar projections from a single view. In this paper, some of the recent methods using depth maps are introduced. However, if more information is required, there are many other interesting methods to analyze [94,95,96,97].

In [98], the authors propose the use of sequences of depth maps for action recognition, which provide additional body shape and motion information. In their approach, in order to make use of the additional body shape and motion information from depth maps, they generate Depth Motion Maps (DMM) by projecting depth maps into three ortoghonal Cartesian planes and accumulating global activities through entire video sequences. Then, a good characterization of the local appearance and shape on DMM is achieved with HOG, Histrogram of Oriented Gradients. HOG descriptors extracted from depth motion map of each projection view (front, top, side) are combined as DMM-HOG, which is used to represent the entire action video sequences. This DMM-HOG descriptor is the input to a linear SVM classifier which is used to make the recognition.

A new descriptor for activity recognition from videos obtained with a depth sensor is presented in [99], called the histogram of oriented 4D surface normals (HON4D). In order to capture the complex joint shape-motion cues at the pixel level, the authors use a histogram to describe depth sequence, which captures the distribution of the surface normal orientation in 4D space of time, depth and spatial coordinates. Instead of concatenating features [100], their histogram, as it operates in 4D space, captures the distribution of the changing shape and motion cues along with their correlation. The histogram is built by creating 4D projectors that represent the possible directions of the 4D normal and, as the descriptor is a representation for the entire sequence, it is robust against noise and occlusion, unlike other methods [101]. To quantize the 4D space, they use the vertices of a polychoron to get a more discriminative quantification.

In [102], the authors present a two-layer Bag-of-Visual-Words (BoVW) model. First, they delete background clutter, so background noise is removed. In addition, foreground noise disturbances are eliminated by jointly using motion and shape information. To distinguish similar actions, motion-based STIPs (spatial-temporal interest points) and shape based STIPs are detected. They use 3DLSK, first mentioned in [103], to describe local structures of motion-based STIPs, and, in order to fit better to depth data and its lack of texture or scale changes effects, they propose a multi-scale 3DLSK (M3DLSK). On the other hand, to capture spatial-temporal relationships among STIPs, they extract a spatial-temporal vector (STV) descriptor for each STIP to distinguish between different actions. Fusing both descriptors, M3DLSK and STV, a feature representation able to capture local and global motion and shape is achieved.

Satyamurthi et al. [104] propose the use of depth motion maps projected on multiple directions, multi-directional projected depth motion map (MPDMM), based on depth motion maps [96,98]. The proposed approach can be separated in three key components. First, they propose to extract features by converting the video sequences into frames using multi-directional projected DMM. The input 3D depth action video is projected into a set of 2D maps according to a set of planes and directions. After calculating the motion energy of each projected map, this is concatenated through entire video sequences to get the MPDMM model. Second, features are extracted from MPDMM model, on the basis of conventional texture-based Local Binary Patterns (LBP) descriptors [105]. The MPDMM image is processed with the LBP technique by thresholding the neighborhood of each pixel and outputting the result as a series of binary numbers that are then used as a statistical measure forming a histogram. Third, the kernel-based Extreme Learning Machine (ELM) [106] with a radial basis function kernel is applied to perform the classification.

In Table 2, a summary of the explained depth information based methods is presented.

### 2.3. Deep Learning Based Methods

After being a breakthrough in image classification, it was a matter of time to start using deep learning for video-based activity recognition. Although great advances have been made and state-of-the-art results have been achieved, the level of image classification has not been reached yet.

In 2014, a paper was released [110] encouraged by the results of *Convolutional Neural Networks* (CNNs) [111] for image recognition problems [112,113,114,115]. Using a 1M videos dataset, they studied different ways for extending the connectivity of a CNN in a time domain in order to take advantage of local spatio-temporal information. They proposed three connectivity patterns: Early Fusion, Late Fusion and Slow Fusion. The Early Fusion extension combines information across an entire time window immediately on the pixel level. The Late Fusion model places two separate single-frame networks with shared parameters a distance of 15 frames apart and then merges the two streams in the first fully connected layer. This way motion can not be detected until the fully connected layer, which compares both outputs to compute global motion. The Slow Fusion model slowly fuses temporal information throughout the network such that higher layers get access to progressively more global information in both spatial and temporal dimensions. For optimization, Downpour Stochastic Gradient Descent [116] is used. The results show that a slow fusion model performs better than the early and late fusion alternatives. They also find out that a single-frame model already displays very strong performance, suggesting that local motion may not be critically important.

In the same year as the previous paper, another work was published [117] that has been the reference of later publications. Simonyan et al. propose a two-stream Convolutional Neural Network architecture that incorporates spatial and temporal networks. Videos can naturally be decomposed into spatial and temporal components. The spatial part provides information about scenes and objects of the video, taking as input a single frame. Nevertheless, the temporal part, which consists of stacked optical flow vectors, shows the movement of the observer (the camera) and the objects in the form of motion across the frames. This way, the authors divide the architecture into two streams. Each stream is implemented using a deep ConvNet [118]; softmax scores are combined by late fusion using a SVM [119] or averaging. It seems that training a temporal network with optical flow improves the training of just stacked frames as in [110]. However, compared to the shallow representation of [76], there are some things to improve yet.

After these two publications, and taking them as a starting point, deep learning has continued to be used for activity recognition, mainly with Convolutional Neural Networks (CNN) and Long Short-Term Memory (LSTM) [120].

In [121], the authors investigate if recurrent models are effective for tasks involving sequences. They propose a Long-term Recurrent Convolutional Network (LRCN) and demonstrate the value of these models for activity recognition. The LSTM unit they use is as the one described in [122]. Compared to previous models, recurrent convolutional models learn compositional representations in space and time and not just assume a fixed visual representation or perform simple temporal averaging for sequential processing. As input, both RGB and optical flow are used and it is observed that the best results are achieved by the weighted scores of both inputs as in [117]. They show that learning sequential dynamics with a deep sequence model improves previous methods that only took into account parameters of the visual domain.

Wang et al. in their work [123] presented very deep two-stream ConvNets in order to improve the results of recent architectures [117] getting closer to image domain deep models. Apart from using two known architectures, GoogLeNet [124] and VGGNet-16 [125], they use 10-frame stacking of optical flow for the temporal network and a single frame image for the spatial network. As the training datasets are small, the model is initialized by pre-training it with ImageNet and, to avoid over-fitting, dropout and data augmentation techniques are used. They proposed two new data augmentation methods: one of them consists of cropping four corners and one center of the images and, in the other, multi-scale cropping is used.

In [126], trajectory-pooled deep-convolutional descriptor (TDD) is introduced, which combines the works of [76,117]. The authors first train two-stream ConvNets and use them as feature extractors to achieve convolutional spatial and temporal feature maps from the learned networks. With the improved trajectories method, a set of point trajectories are detected and, using trajectory pooling, TDD descriptors are created based on normalized convolutional feature maps and these trajectories, as in Equation (5):

(5) $$D\left(T_{k},{\tilde{C}}_{m}^{a}\right)=\sum_{p=1}^{P}{\tilde{C}}_{m}^{a}\left(\bar{\left(r_{m}\times x_{p}^{k}\right)},\bar{\left(r_{m}\times y_{p}^{k}\right)},z_{p}^{k}\right),$$

where $T_{k}$ is a trajectory, ${\tilde{C}}_{m}^{a}$ is a *m*th layer normalized feature map, $\left(x_{p}^{k},y_{p}^{k},z_{p}^{k}\right)$ is the *p*th point position of video coordinates of $T_{k}$ trajectory and $r_{m}$ is the *m*th layer map size ratio, $\bar{\left(\cdot \right)}$ being the rounding operation. Fisher vector representation is used to bring together TDDs over the whole video and, finally, an SVM classifier does the recognition.

Although having some similarities with previous works [110,117], in [127], Tran et al., instead of using 2D convolutions across frames, use 3D convolutions and 3D pooling, propagating temporal information across all the layers in the network. They propose a simple yet effective approach for spatio-temporal feature learning using deep three-dimensional, convolutional networks trained on a large scale supervised video dataset. They show that 3D ConvNets [110,128] with a linear classifier are more suitable for spatio-temporal feature learning than 2D ConvNets and that the model performs even better additionally using hand-crafted features like iDT [76].

In the work by Feichtenhofer et al. [129], the authors add two ideas to the two-stream architecture of [117]. They show that it is important to associate spatial feature maps of a particular area to temporal feature maps for that corresponding region. The spatial and temporal networks are fused at an early level, so, rather than fusing at the softmax layer, they are fused at a convolutional layer. The fusion can be made in different ways and, in [130], Yue-Hei et al. evaluate many other methods to combine two-stream ConvNets over time. The architecture they propose does not increase the number of parameters significantly compared to previous methods and their results are improved by adding also iDT features [76].

Wang et al. also improved the two streams architecture in their work [131], presenting a long-rate temporal structure model, the Temporal Segment Network (TSN). Most of the previous works were not able to incorporate long-range temporal structures, but their model combines a sparse temporal sampling strategy and video-level supervision to enable efficient and effective learning using the whole action video. Another problem they wanted to deal with was over-fitting because, due to the difficulty of collecting data, the available datasets were limited. They use different techniques to avoid the risk of over-fitting: batch normalization [132], dropout [133] and pre-training. The authors also evaluate the model using four different input modalities: optical-flow, warped optical-flow, RGB and RGB difference, the last one inspired by [134].

Bilen et al. [135] presented the concept of dynamic image, which summarizes a video into just a single RGB image by applying rank pooling on the images of a video. This way, image classification CNNs can be used directly, as the input is an image. The idea of reducing the whole video to a single image is taken from [136]. In their experiments, two scenarios were considered: getting a single dynamic figure from a video or getting several dynamic images from each video, the second approach is thought to deal with the lack of training videos. Then, dynamic feature maps are obtained by adding a new temporal layer to the CNN and a pre-trained CaffeNet [137] model is used to initialize the network.

In 2017, Carreira et al. [138] presented a new architecture that uses two different 3D networks for both streams of a two-stream architecture [117], called Two-Stream Inflated 3D ConvNet (I3D). It is based on 2D ConvNet inflation, expanding filters and pooling kernels of very deep image classification ConvNets into 3D, leading to very deep spatio-temporal classifiers and making it possible to learn spatio-temporal feature extractors from videos. In basic two-stream architectures the spatial stream is formed by single frames; however, in I3D, the spatial stream input consists of frames stacked in time dimension. Apart from the new model, the main contribution of this paper is a new dataset for action recognition, the Kinetics Human Action Video dataset, which is two orders of magnitude larger than previous datasets with 400 actions and more than 400 clips per action collected from YouTube. They also showed that, when pre-training on Kinetics, results of I3D models are improved.

Later in 2018, [139] improved the performance of [121] by using lower spatial resolution and longer clips to keep the complexity of networks tractable while dealing with the inability to capture long range temporal information. They consider space-time convolutional neural networks [127,128,140] and study architectures with long-term temporal convolutions (LTC), which are used to learn video representations. As in [121], different low-level representations are studied: RGB and optical flow. Their experiments confirm the advantage of motion-based representations and highlight the importance of good quality motion estimation for learning efficient representations for human action recognition.

Ullah et al. [141] proposed an action recognition method by processing the video data using convolutional neural networks (CNN) and deep bidirectional LSTM (DB-LSTM) networks [142]. On the one hand, in order to reduce complexity and redundancy, deep features are extracted from every six frames of a video using pre-trained AlexNet [112]. Then, the sequential information among frame features is learned using an DB-LSTM network, where multiple layers are stacked together in both forward pass and backward pass of DB-LSTM to increase its depth. The video is analyzed in N chunks and N depends on processing time interval T. The final output is the combination of small chunks outputs. As the video is processed and features are analyzed for a certain time interval, the proposed method is able to learn long sequences and recognize actions in long videos.

Wang et al. [143] proposed a discriminative pooling based on the idea that, among the frames, not all of them have the same importance and a few are those that provide characteristic information about the action [144]; some of the features in one sequence are indeed useful, while the rest are not. Taking all the CNN features as positive (containing good and bad features) and the known background or noisy frames as negative, a nonlinear hyperplane that differentiates the discriminative features from the rest is learned to make the separation. The decision boundary of the classifier thus learned is then used as a descriptor for the entire video sequence, which they call the SVM Pooled (SVMP) descriptor. Thus, they formulate an efficient solver that learns these hyperplanes per video and the corresponding action classifiers over the hyperplanes. This pooling scheme is end-to-end trainable within a deep framework.

The authors of [145] presented the first end-to-end convNets which admit videos of arbitrary size and length. After seeing that 3D convolutional networks have achieved good results in action recognition, they decided to delete two of the requirements that existing convNets had: fixed size and length input videos were required, which reduce the quality of video analysis. Basically, each video is decomposed into spatial and temporal shots and, for both pieces of information, the same process is computed. A spatial temporal pyramid pooling (STPP) convNet is first used to extract equal-dimensional descriptors from variable-sized frame sequences. Then, a Long Short-Term Memory (LSTM) or a CNN-E model is used to recognize the actions from these descriptors. Finally, both streams (spatial and temporal) are combined by a late fusion.

In Table 3, a summary of the deep learning based methods explained is presented.

Finally, in order to compare the presented techniques briefly, some advantages and disadvantages are presented in Table 4.

## 3. Benchmark Datasets

Although there is not a standard benchmark in activity recognition, there are some datasets that are being considered as reference ones [148]. As it has been mentioned above, due to the complexity of collecting data, the available datasets are limited. In this section, the most used datasets are presented.

### 3.1. UCF-101

UCF101 [89,149] is an action recognition dataset of realistic action videos. It is composed of 13,320 videos with 101 action categories and 27 h of video data. This dataset is an extension of the UCF50 [150] dataset that has 50 action categories.

The videos have been collected from YouTube, making the dataset realistic, and it provides a great variety of videos with different objects, camera motion, background, lighting, viewpoint, etc. Based on those features, videos are gathered into 25 groups (4–7 videos per action in each group) with videos sharing some of the features, as background, for example.

The 101 categories can be divided in five main groups:

1. Human–Object Interaction: twenty categories.
2. Body-Motion Only: sixteen categories.
3. Human–Human Interaction: five categories.
4. Playing Musical Instruments: ten categories.
5. Sports: fifty categories.

### 3.2. HMDB51

HMDB51 [88,151] is another action recognition database that collects videos from various sources, mainly from movies but also from public databases such as YouTube, Google and Prelinger Archives.

It consists of 6849 videos with 51 action categories and a minimum of 101 clips belong to each category. The action categories can be divided as well in five main groups:

1. General facial actions: smile, laugh, chew, talk.
2. Facial actions with object manipulation: smoke, eat, drink.
3. General body movements: cartwheel, clap hands, climb, climb stairs, dive, fall on the floor, backhand flip, handstand, jump, pull up, push up, run, sit down, sit up, somersault, stand up, turn, walk, wave.
4. Body movements with object interaction: brush hair, catch, draw sword, dribble, golf, hit something, kick ball, pick, pour, push something, ride bike, ride horse, shoot ball, shoot bow, shoot gun, swing baseball bat, sword exercise, throw.
5. Body movements for human interaction: fencing, hug, kick someone, kiss, punch, shake hands, sword fight.

Apart from the action label, other meta-labels are indicated in each clip. These labels provide information about some features describing properties of the clip, such as camera motion, lighting conditions, or background. As videos are taken from movies or YouTube, the variation of features is high and that extra information can be useful. In addition, the quality of the videos has been measured (*good*, *medium*, *bad*), and they are rated depending on whether body parts vanish while action is executed or not.

### 3.3. Weizmann

Before the two previous databases were created, many methods used the Weizmann [152] database published by [64] to evaluate the performance of their contributions. It provides 90 low-resolution (180 × 144, deinterlaced 50 fps) video sequences. These clips show 10 different actions performed by nine different people. These are the actions that appear in the database: *run, walk, skip, jumping-jack (jack), jump-forward-on-two-kegs (jump), jump-in-place-on-two-legs (pjump), side-gallop (side), wave-two-hands (wave2), wave-one-hand (wave1)* and *bend*. Background and the viewpoint are statics.

### 3.4. MSRAction3D

In 2010, as there was no public benchmark database, the authors in [107] published the database called MSRAction3D [153] which provided the sequences of depth maps captured by a depth camera. The dataset contains twenty actions: *high arm wave, horizontal arm wave, hammer, hand catch, forward punch, high throw, draw x, draw tick, draw circle, hand clap, two hand wave, side-boxing, bend, forward kick, side kick, jogging, tennis swing, tennis serve, golf swing, pick up* and *throw*. Seven different individuals performed each action three times, facing the camera during the performance. The depth maps have a size of 640 × 480 and they were captured at about 15 frames per second (fps) by a depth camera with infra-red light structure.

### 3.5. ActivityNet

The authors of [154] presented in 2015 the ActivityNet [155] database. It is composed of 203 different classes with an average of 137 videos per class and a total of 648 video hours. The videos were obtained from online video sharing sites and they are around 5–10 min long. Half of the videos are in HD resolution (1280×720) and most of them have a frame rate of 30 fps.

The aim of this database is to collect activities of humans daily life and it has a hierarchical structure, organizing the activities according to social interactions and where they take place.

### 3.6. Something Something

Later, in 2017, the authors of [156] introduced the “Something Something” [157] dataset. The first version of the database consists of 108,499 videos belonging to 174 different labels with 23,137 distinct object names. The length of the videos variate between 2 and 6 s and they have a height of 100 px and variable width. Labels are textual descriptions such as “Putting *something* next to *something*” where *something* refers to an object name. This database is already split into train, validation and test, containing 86,017, 11,522 and 10,960 videos, respectively.

However, there has been a second release of the dataset and now it contains 220,847 videos, 168,913 for the training set, 24,777 for the validation set and 27,157 for the test set. The number of labels remains the same, but there are additional object annotations now. Moreover, the pixel resolution has increased from 100 px to 240 px.

### 3.7. Sports-1M

In [110], Karpathy et al. presented a new database, Sports-1M [158], which contains 1,133,158 video URLs with 487 automatically annotated different labels. YouTube Topics API was used to do the annotation. There are around 1000–3000 videos per class and some of them, nearly the 5%, are labelled with more than one class.

Nowadays, the YouTube-8M [159] dataset is also available and the Sports-1M dataset is included in it. This dataset is composed of videos from 3862 labels and it contains 350,000 h of video. In this case, each video has an average of three labels.

### 3.8. AVA

The authors of [160] presented AVA [161], a video dataset of spatio-temporally localized Atomic Visual Actions. This dataset consists of 430 movie clips of 15 min length annotated with 80 actions (14 poses, 17 person–person, 49 person–object). There are 386,000 labelled segments, 614,000 labelled bounding boxes and 81,000 person tracks, with a total of 1.58M labelled actions, with multiple labels per person occurring frequently.

Every person of the scene is localized by a bounding box and labels are assigned according to the action performed by the actor. Each scene can have more than a label, one of them corresponds to the actor’s pose and additional labels which correspond to person–object or person–person interactions can be assigned. A frame containing more than one actor is labelled separately for each person of the scene.

To finish, in Table 5, a summary of the explained datasets is introduced, in order to present the information more clearly.

## 4. Results

To better analyze the explained methods and the contributions of each one of them, the results obtained for mentioned datasets are compared. For each method, the achieved accuracy values for different datasets are shown, together with the reference to the original article where they have been proposed.

On the one hand, in Table 6, results for depth information based methods can be observed. These methods use the MSRAction3D as benchmarks because the input they need is different from other models. Regarding the methods used with the MSRAction3D database, the best result of the presented methods is achieved by [102], as it can be seen in Table 6.

On the other hand, most of the hand-crafted feature methods use the Weizmann dataset as a benchmark. However, some of the presented models work with both UCF101 and HMDB51 datasets, which are used as benchmarks in deep learning methods. Thus, in Table 7, the obtained accuracy values can be observed, together for deep learning and hand-crafted methods.

As it can be seen in Table 7, the Two-Stream I3D method [138], pre-trained with the Kinetics dataset, provides the best result for the UCF101 dataset. For HMDB51, the best result is achieved by the DB-LSTM model [141] and, among those who have tested with the Weizmann database, the best value is given by the method presented in [63].

## 5. Discussion

After reading the previous sections, the researchers could ask themselves which are the most promising lines of research in the field of action recognition in videos, or where it is more likely to get a higher return for the invested effort.

For people just interested in applying an existing method to their data, or in minimal modifications or customizations, some authors of the presented methods have made their code available that can be used. These implementations are indicated in Table 8. Among them, the methods explained in [138,141] provide the best results.

For researchers interested in developing new methods or in deep modifications of current ones, deep learning looks like the way to go, although the computations costs could be forbidding.

Some researchers could have the resources to generate new datasets similar to those presented in this paper. They need to have in mind several decisions: will the videos have the same resolution and/or length and will they be recorded with the same background? We advocate for datasets covering different kinds of videos, with that information present in the metadata and with enough samples of each type. Anyway, if the researcher resources are somewhat limited, which is usually the case, it is advisable to focus on just one type of video. All the technical information about the sensor might appear in the metadata, as well as lighting conditions or any information of interest. If depth sensors are used, high and low resolution of the depth data could be provided. Processing of depth data can be computationally expensive, and other researchers using that dataset could benefit from access to a standard low resolution version of that data.

The task of labeling the database samples can be eased with the help of some tools. While just providing a global label for a video does not require a great deal of effort, the video database curators could choose to gather information about individual frames in the videos. There are several tools that could be useful in this task, like Sloth [163], LabelMe [164,165] or LabelBox [166].

It is difficult to predict the future development of this area, but, at least in the short term, the overall tendency in machine learning is going towards massive data, computationally expensive algorithms and dedicated hardware. It is expected that the price of depth sensors will keep a descending curve, as well as the cost of hardware in general. The main challenges are expected to be twofold: for the researchers developing new methods, those related to the storage and processing of massive databases, and for developers integrating the methods into software solutions, those related to a fast classification time.

## 6. Conclusions

In this paper, different methods for video activity recognition have been presented. Several models have been explained showing the development of recent years. Likewise, several databases used to evaluate the performance of the models have been introduced. The results have been shown together in a table in order to compare the methods presented correctly.

Due to the extent width of the subject, there are many more models that have not been mentioned in this document. Even so, an attempt has been made to show a current state-of-the-art by presenting different techniques to deal with the problem. To sum up, through this document, we have tried to show the relevance and current situation of video-based activity recognition.

Video-based activity recognition, as it has been mentioned before, is more complicated than static image classification and this is also reflected in the results obtained so far. However, since deep learning is still being exploited, in the near future, this task may become easier to perform and current results may be improved using some deep learning techniques.

## Figures and Tables

**Table 1:** Summary of methods using hand-crafted motion features.

	YEAR	SUMMARY	DATASET
Bobick et al. [47]	2001	Use of motion-energy image (MEI) and motion-history image (MHI).	-
Schuldt et al. [49]	2004	Use of local space-time features to recognize complex motion patterns.	KTH Action [49]
Niebles et al. [55]	2007	Use of a hybrid hierarchical model, combining static and dynamic features.	Weizmann [64]
Laptev et al. [42]	2008	Use of spatio-temporal features and extend spatial pyramids to spatio-temporal pyramids.	KTH Action [49] Hollywood [42]
Chen et al. [63]	2009	Use of HOG for human pose representations and HOOF to characterize human motion.	Weizmann [64] Soccer [83] Tower [63]
Chaudhry et al. [46]	2009	Use of HOOF features by computing optical flow at every frame and binning them according to primary angles.	Weizmann [64]
Lertniphonphan et al. [45]	2011	Use of a motion descriptor based on direction of optical flow.	Weizmann [64]
Wang et al. [76]	2013	Use of camera motion to correct dense trajectories.	HMDB51 [88] UCF101 [89] Hollywood2 [90] Olympic Sports [91]
Akpinar et al. [81]	2014	Use of a generic temporal video segment representation, introducing a new velocity concept: Weighted Frame Velocity.	Weizmann [64] Hollywood [42]
Kumar et al. [15]	2016	Use of a local descriptor built by optical flow vectors along the edges of the action performers.	Weizmann [64] KTH Action [49]
Sehgal, S. [87]	2018	Use of background subtraction, HOG features and BPNN classifier.	Weizmann [64]

**Table 2:** Summary of depth information based methods.

	YEAR	SUMMARY	DATASET
Yang et al. [98]	2012	Use of Depth Motion Maps (DMM), combining them with HOG descriptors.	MSRAction3D [107]
Oreifej et al. [99]	2013	Use of histogram of oriented 4D surface normals (HON4D) descriptor.	MSRAction3D [107] MSRGesture3D [108] 3D Action Pairs [99]
Liu et al. [102]	2018	Use of a two-layer BoVW model, using motion-based and shape-based STIPs to distinguish the action.	MSRAction3D [107] UTKinect-Action [109] MSRGesture3D [108] MSRDailyActivity3D [100]
Satyamurthi et al. [104]	2018	Use of multi-directional projected depth motion maps (MPDMM).	MSRAction3D [107] MSRGesture3D [108]

**Table 3:** Summary of deep learning based methods.

	YEAR	SUMMARY	DATASET
Karpathy et al. [110]	2014	Use of different connectivity patterns for CNNs: early fusion, late fusion and slow fusion.	Sports-1M [110] UCF101 [89]
Simonyan et al. [117]	2014	Use of a two-stream CNN architecture, incorporating spatial and temporal networks.	UCF101 [89] HMDB51 [88]
Donahue et al. [121]	2015	Use of a Long-term Recurrent Convolutional Network (LRCN) to learn compositional representations in space and time.	UCF101 [89]
Wang et al. [123]	2015	Use of very deep two-stream convNets, using stacked optical flow for temporal network and a single frame image for spatial network.	UCF101 [89]
Wang et al. [126]	2015	Use of trajectory-pooled deep-convolutional descriptor (TDD).	UCF101 [89] HMDB51 [88]
Tran et al. [127]	2015	Use of deep 3D convolutional networks, which are better for spatio-temporal feature learning.	UCF101 [89]
Feichtenhofer et al. [129]	2016	Use of two-stream architecture associating spatial feature maps of a particular area to temporal feature maps of that region and fusing the networks at an early level.	UCF101 [89] HMDB51 [88]
Wang et al. [131]	2016	Use of Temporal Segment Network (TSN) to incorporate long-range temporal structures avoiding overfitting.	UCF101 [89] HMDB51 [88]
Bilen et al. [135]	2016	Use of image classification CNNs after summarizing the videos in dynamic images.	UCF101 [89] HMDB51 [88]
Carreira et al. [138]	2017	Use of two-stream Inflated 3D ConvNet (I3D), using two different 3D networks for both streams of a two-stream architecture.	UCF101 [89] HMDB51 [88]
Varol et al. [139]	2018	Use of space-time CNNs and architectures with long-term temporal convolutions (LTC), using lower spatial resolution and longer clips.	UCF101 [89] HMDB51 [88]
Ullah et al. [141]	2018	Use of CNNs to reduce complexity and redundancy and deep bidirectional LSTM (DB-LSTM) to learn sequential information among frame features.	UCF101 [89] HMDB51 [88] YouTube actions [146]
Wang et al. [143]	2018	Use of a discriminative pooling, taking into account that just a few frames provide characteristic information about the action.	HMDB51 [88]
Wang et al. [145]	2018	Use of convNets which admit videos of arbitrary size and length, using first a STPP and a LSTM (or CNN-E) then.	UCF101 [89] HMDB51 [88] ACT [147]

**Table 4:** Advantages and disadvantages of presented techniques.

	Advantages	Disadvantages
Hand-crafted motion features	- There is no need of a large amount of data for training. - It is simple and unambiguous to understand the model and analyze and visualize the functions. - The features used to train the model are explicitly known.	- Usually these features are not robust. - They can be computationally intensive due to the high dimensions. - The discriminative power is usually low.
Depth information	- The 3D structure information of the image that depth sensors provide is used to recover postures and recognize the activity. - The skeletons extracted from depth maps are precise. - Depth sensors can work in darkness.	- Depth maps have no texture, making it difficult to apply local differential operators. - The global features can be unsettled because depth maps may contain occlusions.
Deep Learning	- There is no need of expert knowledge to get suitable features, reducing the effort of feature extraction. - Instead of designing them manually, features are automatically learned through the network. - Deep neural networks can extract high-level representation in deep layer, making it more suitable for complex tasks.	- Need to collect massive data, consequently there is a lack of data sets. - Time consuming. - Problem of models capability of generalization.

**Table 5:** Summary of the presented datasets.

	# Classes	# Videos	# Actors	Resolution	Year
Weizmann	10	90	9	180 × 144	2005
MSRAction3D	20	420	7	640 × 480	2010
HMDB51	51	6849	-	320 × 240	2011
UCF50	50	6676	-	-	2012
UCF101	101	13,320	-	320 × 240	2012
Sports-1M	487	1,133,158	-	-	2014
ActivityNet	203	27,801	-	1280 × 720	2015
Something Something	174	220,847	-	__ (Variable width) × 240	2017
AVA	80	430	-	-	2018

**Table 6:** Obtained accuracies for the benchmark dataset with depth information based methods.

	METHOD	MSRAction3D
DS	DMM-HOG [98]	85.52%
	HON4D [99]	88.89%
	M3DLSK+STV [102]	**95.36%**
	MPDMM [104]	94.8%

**Table 7:** Obtained accuracies for the benchmark datasets with hand-crafted methods and deep learning methods.

	METHOD	UCF101	HMDB51	Weizmann
Hand-crafted	Hierarchical [55]	-	-	72.8%
	Far Field of View [63]	-	-	**100%**
	HOOF NLDS [46]	-	-	94.4%
	Direction HOF [45]	-	-	79.17%
	iDT [76]	-	57.2%	-
	iDT+FV [76]	85.9%	57.2%	-
	OF Based [81]	-	-	90.32%
	Edges OF [15]	-	-	95.69%
	HOG features [87]	-	-	99.7%
Deep learning	Slow Fusion CNN [110]	65.4%	-	-
	Two stream (avg) [117]	86.9%	58.0%	-
	Two stream (SVM) [117]	88.0%	59.4%	-
	IDT+MIFS [162]	89.1%	65.1%	-
	LRCN (RGB) [121]	68.2%	-	-
	LRCN (FLOW) [121]	77.28%	-	-
	LRCN (avg, 1/2-1/2) [121]	80.9%	-	-
	LRCN (avg, 1/3-2/3) [121]	82.34%	-	-
	Very deep two-stream (VGGNet-16) [123]	91.4%	-	-
	TDD [126]	90.3%	63.2%	-
	TDD + iDT [126]	91.5%	65.9%	-
	C3D [127]	85.2%	-	-
	C3D + iDT [127]	90.4%	-	-
	TwoStreamFusion [129]	92.5%	65.4%	-
	TwoStreamFusion+iDT [129]	93.5%	69.2%	-
	TSN (RGB+FLOW) [131]	94.0%	68.5%	-
	TSN (RGB+FLOW+WF) [131]	94.2%	69.4%	-
	Dynamic images + iDT [135]	89.1%	65.2%	-
	Two-StreamI3D [138]	93.4%	66.4%	-
	Two-StreamI3D, pre-trained [138]	**97.9%**	80.2%	-
	LTC (RGB) [139]	82.4%	-	-
	LTC (FLOW) [139]	85.2%	59.0%	-
	LTC(FLOW+RGB) [139]	91.7%	64.8%	-
	LTC(FLOW+RGB)+iDT [139]	92.7%	67.2%	-
	DB-LSTM [141]	91.21%	**87.64%**	-
	Two-Stream SVMP(VGGNet) [143]	-	66.1%	-
	Two-Stream SVMP(ResNet) [143]	-	71.0%	-
	Two-Stream SVMP(+ iDT) [143]	-	72.6%	-
	Two-Stream SVMP(I3D conf) [143]	-	83.1%	-
	STPP + CNN-E (RGB) [145]	85.6%	62.1%	-
	STPP + LSTM (RGB) [145]	85.0%	62.5%	-
	STPP + CNN-E (FLOW) [145]	83.2%	55.4%	-
	STPP + LSTM (FLOW) [145]	83.8%	54.7%	-
	STPP + CNN-E (RGB+FLOW) [145]	92.4%	70.5%	-
	STPP + LSTM (RGB+FLOW) [145]	92.6%	70.3%	-

**Table 8:** Available code for presented methods.

METHOD	YEAR	PAPER	CODE
Deep Learning	2018	Video representation learning using discriminative pooling [143]	SVMP https://github.com/3xWangDot/SVMP
Deep Learning	2018	Action Recognition in Video Sequences using Deep Bi-Directional LSTM With CNN Features [141]	Bi-directional LSTM https://github.com/Aminullah6264/BidirectionalLSTM
Deep Learning	2018	Long-term temporal convolutions for action recognition [139]	LTC https://github.com/gulvarol/ltc
Deep Learning	2017	Quo vadis, action recognition? A new model and the Kinetics dataset [138]	Two-Stream I3D https://github.com/deepmind/kinetics-i3d
Deep Learning	2016	Dynamic image networks for action recognition [135]	Dynamic images https://github.com/hbilen/dynamic-image-nets
Deep Learning	2016	Temporal segment networks: Towards good practices for deep action recognition [131]	TSN https://github.com/yjxiong/temporal-segment-networks
Deep Learning	2016	Convolutional two-stream network fusion for video action recognition [129]	Two-Stream Fusion https://github.com/feichtenhofer/twostreamfusion
Deep Learning	2015	Learning spatiotemporal features with 3D convolutional networks [127]	C3D https://github.com/facebook/C3D
Deep Learning	2015	Action recognition with trajectory-pooled deep-convolutional descriptors [126]	TDD https://github.com/wanglimin/tdd/
Deep Learning	2015	Towards good practices for very deep two-stream convNets [123]	Very deep Two-Stream convNets https://github.com/yjxiong/caffe/tree/action_recog
Depth information	2013	HON4D: Histogram of oriented 4D normals for activity recognition from depth sequences [99]	HON4D http://www.cs.ucf.edu/~oreifej/HON4D.html
Hand-crafted motion features	2013	Action Recognition with Improved Trajectories [76]	Improved Trajectories http://lear.inrialpes.fr/~wang/improved_trajectories
